# Endoscopic reversal of roux-en-Y gastric bypass prevents worsening of nutritional outcomes in patients with severe malnutrition

**DOI:** 10.3389/fgstr.2023.1212844

**Published:** 2023-07-14

**Authors:** Nirjhar Dutta, Adam W. Scott, Nicholas A. Marka, Eric S. Wise, Stuart K. Amateau

**Affiliations:** ^1^ Division of Hospital Medicine, University of Minnesota, Minneapolis, MN, United States; ^2^ University of Minnesota Medical School, Minneapolis, MN, United States; ^3^ Clinical and Translational Sciences Institute, University of Minnesota, Minneapolis, MN, United States; ^4^ Department of Surgery, University of Minnesota, Minneapolis, MN, United States; ^5^ Division of Gastroenterology, Hepatology and Nutrition, University of Minnesota, Minneapolis, MN, United States

**Keywords:** endoscopic reversal, roux-en-Y gastric bypass, severe protein caloric malnutrition, weight loss, artificial nutrition, per oral nutrition, total parenteral nutrition, tube feed

## Abstract

Roux-en-Y gastric bypass (RYGB) can precipitate protein-calorie malnutrition and micronutrient deficiencies. Sonographically guided endoscopic reversal (ER) *via* deployment of a stent from the gastric pouch to the remnant stomach in RYGB anatomy has emerged as a novel option for increasing both intestinal transit time and absorptive surface area. In this investigation, short-term nutritional outcomes after ER of a RYGB in patients (age ≥ 18) with severe protein-calorie malnutrition from a single academic health center in Minneapolis, Minnesota over a seven-year period (2015-2021) were retrospectively reviewed pre-procedurally, and at six and twelve months post-procedurally. 17 patients underwent ER for severe protein-calorie malnutrition, or dependence on tube feeds (TF) or total parenteral nutrition (TPN). At 6 months post-ER, two patients were no longer malnourished and only on oral nutrition; three patients were liberated from TPN. Laboratory markers of protein-calorie malnutrition, renal function, and micronutrients were not significantly different at six- or twelve-month follow-up (P > 0.05). In all patients, access to the gastric remnant was maintained *via* stent placement through the gastric pouch or proximal Roux limb throughout the study period and no complications were noted after ER. Despite the small sample size, this investigation revealed that ER of RYGB may prevent progressive deleterious weight loss, and worsening macro- and micro-nutrient deficiencies, though improvement in weight and nutritional parameters was not observed. Overall, ER was found to be a nuanced and safe, advanced technique useful for when remnant access is desired in RYGB patients.

## 1 Introduction

Bariatric surgeons in the United States perform over 252,000 bariatric procedures every single year (1). The Roux-en-Y gastric bypass (RYGB) constitutes one of the most common and effective bariatric procedures performed in the treatment of obesity (1, 2). During a RYGB, the receptive stomach is downsized to a small pouch the size of an egg (10-35ml) (3). The remaining portion of the stomach is no longer receptive to food and anastomosed to a proximal portion of the small intestine, the biliopancreatic limb. The small intestine itself is divided, with the lower part rerouted to the newly created stomach pouch. This rerouted portion of the small intestine is known as the “Roux limb” or the “alimentary limb.” The intestinal rearrangement bypasses a significant portion of the small bowel surface area. This leads to a common channel of small bowel where biliary digestive juices mix with food to aid in digestion (2). In rare cases, however, a RYGB can precipitate protein-calorie malnutrition and micronutrient deficiencies. Supplementation of micronutrients is necessary after RYGB, with severe malnutrition estimated to occur in 4% of patients following a Roux-en-Y gastric bypass surgery (4, 5).

Medical management of malnutrition after a RYGB often entails utilizing supplemental sources of nutrition (4). Tube feeds (TF; *via* placement of a tube in the remnant stomach, or jejunum), total parenteral nutrition (TPN) and appetite stimulants are first line interventions to mitigate chronic malnutrition following a complicated RYGB (6). When these approaches fail or are no longer sustainable to augment a patient’s nutritional status, a surgical reversal of the Roux-en-Y anatomy may be warranted. Surgical RYGB reversals, however, are not without risk, and carry an overall 30-day complication rate of 29% (7). Reported complications include gastrogastric anastomotic leak, sepsis, and bleeding requiring transfusion. Additionally, it has been reported that as many as 57% of patients can develop *de novo* gastroesophageal reflux disease (GERD) following a RYGB reversal surgery (8).

**Figure 1:**
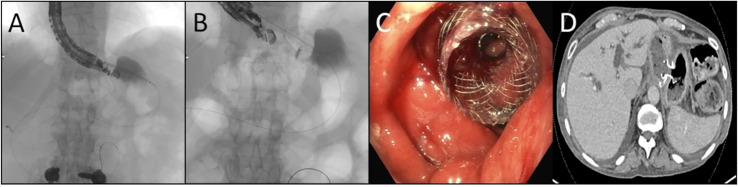
Endoscopic Reversal of Roux-en-Y **(A)** The remnant stomach is localized by endosonography and subsequently accessed by passing a catheter preloaded with a stent across the pouch or Roux as well as the wall of the remnant using electrocautery **(B)** Using combination of ultrasonography, fluoroscopy, and endoscopy the stent is deployed with each bell expanding in the desire lumen **(C)** Following deployment the covered lumen apposing metal stent is found well seated with the remnant seen across the saddle which may be subsequently dilated **(D)** Axial computed tomographic image demonstrating the stent positioned across the pouch and remnant.

Endoscopic RYGB reversal (ER; **Figure 1**) offers a promising, less-invasive alternative to surgical inventions for chronically malnourished patients in the setting of a RYGB (9). Recent advancements in endoscopy have led to the concept of “endoscopic gastrointestinal anastomoses” utilizing lumen-apposing metal stents (LAMS). After establishing wire access to the excluded “remnant stomach,” LAMS are deployed using endoscopic ultrasound from the easily accessible gastric pouch (gastrogastrostomy), or proximal Roux limb (jejunogastrostomy) (9). Access through the gastrogastrostomy is often favored, however, given the thickness and durability of each lumen, anatomy at may not allow for LAM deployment through the gastric pouch, which may be the case due to the distance between structures or intervening bowel. In these instances, the jejunum of the Roux is next evaluated for an appropriate window (9, 10). Endoscopic reversals using LAMS-based anastomoses have been useful adjuncts for antegrade access to the biliary tree for endoscopic retrograde cholangiopancreatography (ERCP) and other endoscopic biliary interventions in patients with Roux-en-Y anatomy. This anastomosis, however, can also be used as an outlet to increase protein-calorie absorptive surface area, or as a conduit to place a percutaneous gastrostomy tube in the remnant stomach. The increased surface area is uniquely valuable in RYGB patients with chronic malnutrition, making them poor surgical candidates, or for patients for whom significant adhesive disease may preclude minimally invasive surgical access to the remnant stomach (9). To date, ER of RYGB anatomy using minimally invasive endoscopic techniques has been shown to be technically feasible (9). There has, however, only been limited data on the short- and long-term outcomes of patients following the ER procedure (9–12). These case reports have shown ER outcomes to have an acceptable safety profile in the short term (9). The aim of this case series is to report six- and twelve-month nutritional and procedural outcomes in 17 patients who underwent ER of RYGB for severe protein calorie malnutrition (SPCM) or who were dependent on TF or TPN, as an alternative to a surgical reversal of their RYGB anatomy.

## 2 Methods

This case series retrospectively reviewed adult patients (age ≥ 18) that underwent ER of RYGB for a primary indication of SPCM or were dependent on TF or TPN for nutrition between March 2015 and May 2022 at the University of Minnesota Medical Center by manual chart review of all patients who underwent ER. Patients that had a RYGB reversal for other indications outside of SPCM (e.g. access for ERCP) or those that opted out of research were excluded. In this study, SPCM was defined as progressive weight loss despite modified high protein diets. This study was approved by the University of Minnesota Institutional Review Board and was exempt from consent due to retrospective use of routine clinical data.

Patient demographics, including race, sex, age, insurance status, nutrition status, mode of nutrition, weight, and BMI were collected by retrospective chart review. Primary nutrition mode was categorized as per oral (PO), tube feed (TF), or total parenteral nutrition (TPN). Nutrition status and mode of nutrition were determined from clinic notes authored by a registered dietitian or gastroenterologist. The reviewed patient’s malnutrition status was classified as malnourished if the patient was noted to have SPCM, or in the event that the clinic notes indicated a patient to be diagnosed with malnutrition but did not indicate the severity of the patient’s malnutrition, the patient’s “undifferentiated malnutrition” was sufficient to be identified as malnourished for the purposes of this study.

Patients’ nutrition status, mode of nutrition, weight, and BMI were accessed at four different time points throughout the study: at 12 months pre-ER, at 6 months pre-ER, at 6 months post-ER, and at 1-year post-ER. Data that were most proximal to the four listed time points were utilized, with no data point used exceeding 90 days from the targeted pre- and post-ER time point.

Key lab values to ascertain the patient’s malnutrition status outside of BMI were also reviewed and included albumin, pre-albumin, calcium, magnesium, phosphorus, PTH, vitamin D, B1, folate, B12, vitamin A, vitamin E, zinc, iron, copper, and transferrin. The malnutrition labs were documented at 6 months pre-ER and 6 months post-ER. Labs that were most proximal to the 6-month pre-ER and 6-month post-ER time points were utilized, with no lab exceeding 90 days from the targeted pre and pos-ER time. All patients experienced malnutrition prior to their ER. In all cases the RYGB occurred greater than 3 years prior to ER and was performed at a different health care institution than the academic health center that performed the patient’s ER. This prevented the analysis of laboratory data immediately prior to or after the initial RYGB surgery, presenting one limitation of the present study.

### 2.1 Statistical analysis

Participant weight and lab values were summarized as medians and interquartile ranges (IQR). Comparisons between lab values at 6 months pre-baseline and 6 months post-procedure were conducted using a Wilcoxon Rank-Sum Test. Overall difference in patient weight between time points was assessed using Friedman’s test. A Friedman’s test was used to compare the medians of all values at the time-points considered, using interquartile range (IQR) as the measure of central tendency. All analyses were conducted at the 0.05 significance level using the R software version 4.2.0 (13).

## 3 Results

Seventeen patients were identified through retrospective chart review as having underwent an ER between March 2015-May 2022 at the University of Minnesota Medical Center. All 17 patients underwent technically successful ER by means of gastrointestinal anastomosis. During this time frame there were no unsuccessful ER attempts. All patients that underwent ER in this case review did so by means of a gastrogastrostomy (n=13; gastric pouch to gastric remnant) or jejunogastrostomy (n=4; proximal Roux limb to gastric remnant) with an EUS-guided LAMS placement. The most common size of LAMS deployed was a 15x10mm that was subsequently post-dilated between 10-15mm. The average duration of the ER procedure was 36 minutes with a range of 13-66 minutes. In all of the 17 ER cases, no procedural or post-procedural complications were noted. The median hospital stays for the 17 patients that underwent the ER was 7 hours, with a range of hospital stays between 5 hours-100 days. Additionally, throughout the period of review all patients-maintained access to their gastric remnant following their ER of their RYGB anatomy. During the time period reviewed in the study, no patients had a subsequent surgical reversal after receiving an ER. The individual patient demographic, procedural details, weight, and nutritional outcomes achieved during the study’s time period reviewed throughout this case series are summarized in **Table 1**.

**Table 1:** Individual patient demographics, procedural details, and outcomes.

Patient	Age (year)	Sex	Race	Procedure type	Stent Used	Procedure Duration (minutes)	Procedural Complication	BMI (kg/m^2^) at, baseline	BMI (kg/m^2^) at 6 months post ER	Malnutrition Status at Baseline	Malnutrition Status at 6 months Post ER	Primary Nutrition mode at Baseline	Primary Nutrition Mode at 6 months Post ER
1	44	Male	White	Gastrogastrostomy	10mm x 10mm Axios LAMS	44	No complications	26.2	24.0	Malnourished	Malnourished	PO	PO
2	46	Female	Black	Gastrogastrostomy	Hot 15mmx10mm axios covered LAMS; postdilated to 13.5m	33	No complications	17.9	22.7	Malnourished	Malnourished	TPN	TPN
3	57	Female	White	Jejunogastrostomy	15mmx10mm Axios	33	No complications	26.4	28.8	Malnourished	Not Malnourished	TPN	TPN
4	33	Female	Black	Gastrogastrostomy	Hot Axios 15mmx10mm	38	No complications	23.8	18.0	Malnourished	Malnourished	TF	TF
5	49	Male	White	Gastrogastrostomy	15x10mm LAMS postdilated to 15mm	35	No complications	17.2	16.4	Malnourished	Malnourished	TPN	TF
6	45	Female	White	Jejunogastrostomy	Hot Axios 15x10mm, postdilated to 12mm	28	No complications	23.2	23.7	Malnourished	Malnourished	PO	TPN
7	58	Male	White	Gastrogastrostomy	15mm x 10mm Axios post dilated to 13.5mm	44	No complications	33.2	31.9	Malnourished	Not Malnourished	TF	TF
8	49	Female	White	Gastrojejunostomy	15x10mm covered metal Axios LAMS	48	No complications	23.5	25.1	Malnourished	Malnourished	TPN	PO
9	68	Male	White	Gastrojejunostomy	20x10mm Axios	13	No complications	27.4	25.1	Malnourished	Not Malnourished	PO	PO
10	69	Female	White	Jejunogastrostomy	15x10mm covered LAMS postdilated to 12mm	60	No complications	34.0	42.2	Malnourished	Malnourished	PO	TF
11	51	Female	White	Jejunogastrostomy	15mmx12mm Axios, stent post dilated to12mm	29	No complications	29.0	20.7	Malnourished	Malnourished	TPN	TF
12	54	Female	White	Gastrojejunostomy	Axios 15mm x 10mm	66	No complications	25.2	23.4	Malnourished	Malnourished	TPN	TPN
13	39	Female	Black	Gastrogastrostomy	23x120mm covered EndoMaxx	26	No complications	21.2	25.7	Malnourished	Not Malnourished	TPN	PO
14	55	Female	White	Gastrogastrostomy	15x10mm Axios LAMS postdilated to 10mm	46	No complications	17.9	Not Reported	Malnourished	Malnourished	PO	PO
15	47	Female	White	Gastrojejunostomy	15mm x 10mm Axios postdilated to 12mm	27	No complications	32.1	31.9	Malnourished	Malnourished	TPN	TPN
16	65	Female	White	Gastrogastrostomy	15mm x 10mm Axios post dilated to 13.5mm	34	No complications	17.8	20.4	Malnourished	Malnourished	TF	TF
17	44	Male	White	Gastrojejunostomy	15x10mm Axios LAMS postdilated to 12mm	15	No complications	29.0	28.0	Malnourished	Malnourished	PO	PO

ER, Endoscopic Reversal; BMI, Body Mass Index; TPN, total parenteral nutrition; TF, Tube Feed; PO, Per Os.

The demographic information collected on the 17 patients revealed the median age of these patients at the time of the ER to be 49 [IQR 46-58] years old. Additionally, 71% of all reviewed ER patients were female, and 18% of ER patients identified as being non-white. One patient among the seventeen patients was without health insurance at the time of the procedure.

**Figure 2:**
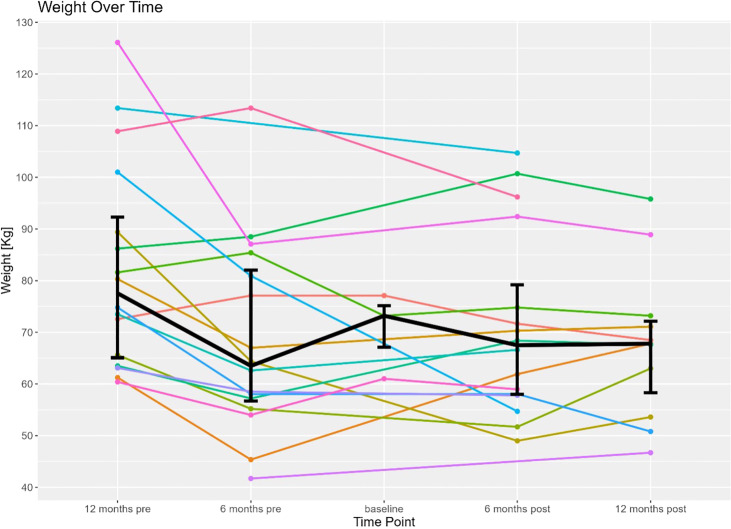
Displays individual weight trends of the 17 patients included in this study 12 months before to 12 months after endoscopic reversal of their RYGB (time of procedure is baseline). Bolded line represents the mean weight of the patient cohort 12 months before to 12 months after endoscopic reversal.

Prior to the ER all patients had extensive collaboration by various specialist including dietitians, and gastroenterologist to evaluate the best strategy to address the patient’s malnutrition. During the patient’s malnutrition work up, patients were also seen by bariatric surgeons that deemed the patients underlying malnutrition not be due to an underlying structural defect in their RYGB anatomy. At the time of ER (considered the patient’s baseline in this study) all 17 patients had a nutrition status of SPCM, undifferentiated malnutrition, or were dependent on TF or TPN for nutrition. At 6-months post-ER, 15 out of the 17 patients had a nutrition status of SPCM, undifferentiated malnutrition, or were on TF/TPN. Conversely, at 6-months post-ER, 2 of the 17 patients were no longer considered malnourished and were solely on PO nutrition. Of the 17 patients that had an ER, 9 patients had follow-up data available at 12 months post-ER time point. Eight out of the 9 patients had SPCM or undifferentiated malnutrition or were on TF/TPN. One of the 9 patients continued to receive full nutrition by PO at the 12-month period and successfully maintained a nutrition status of being non-malnourished. Data at the 12 months was not available for the second patient that successfully reversed their malnutrition status to non-malnourished at 6 months. The composite nutrition status and weight outcomes for the 17 patients that underwent an ER in this study are shown in **Table 2**. As documented in **Table 2**, the overall nutritional status of the 17 patients undergoing an ER did not improve. It is also demonstrated that the 17 ER patient’s nutritional status, mode of nutrition, and BMI also did not worsen during the period of the study. **Figure 2** shows the weight trend for the 17 reviewed patients from 12 months pre-ER to 12 months post ER. Additionally, “spaghetti” plots demonstrating the trajectory of weight loss prior to, and after ER are shown for the 17-patient cohort.

**Table 2:** Patient Malnutrition Status, Nutrition Mode, and Weight metrics pre-ER of RYGB, at baseline, at 6 months, and 12 months post-ER of RYGB.

	Baseline	6 Months Post-ER	12 Months Post-ER
Malnutrition Status	N=17	N=17	N=9
Severe or undifferentiated or on TF/TPN (n, %)	17 (100%)	15 (88%)	8 (89%)
Moderate	0	0	0
Non-severe	0	0	0
Not malnourished	0	2 (12%)	1 (11%)
Nutrition Route	N=17	N=17	N=9
Oral intake (PO)	6	6	2
Tube feeding (TF)	3	6	3
Total parenteral nutrition (TPN)	8	5	4
Weight/BMI	N=17	N=17	N=9
Weight (Kg, median, IQR)	73.2 (67.1, 75.16)	67.5 (58, 79.2)	67.8 (58.3, 72.15)
BMI (median, IQR)	23.16 (22.13, 24.5)	24.56 (22.2, 29.57)	23.16 (20.2, 27.01)

A panel of laboratory values, which served as surrogates of protein-calorie malnutrition in this study (e.g. albumin), renal function (e.g. creatinine) and micronutrients/vitamins/mineral (e.g. B12, Folate) did not significantly change at six-month follow-up (P>0.05; **Supplementary Table 1**, see **supplementary materials**). There was demonstrable improvement in iron from baseline to 6 months post-ER from 44 (22.5, 79) ug/dL *vs* 34.5 (19, 49.25) ug/dL. Notably, no change in albumin was observed at 6 months and 12 months.

## 4 Discussion

ER is an attractive emerging option in patients that have previously had RYGB that then require interventions for SPCM. While ER has been shown to be technically feasible, there have only been limited reports on the short and long-term outcomes of patients following ER (9–12). This case series retrospectively reviewed 17 patients to better characterize the clinical course and outcomes of patients that undergo an ER of a RYGB in the setting of SPCM requiring supplemental nutrition. The principle finding suggests that ER may blunt the exacerbation of malnutrition, while allowing select patients to be liberated from TPN or TF.

In the twelve months pre-ER until 6 months pre-ER, patients in this study nearly uniformly observed a progressive decline in their overall weight. A RYGB precipitates weight loss for an average of 18 months following the bariatric procedure, which is then usually followed by weight stabilization. Patients in this study that received an ER due to malnutrition, however, all had their initial RYGB greater than 3 years prior to their ER (14). Therefore, the observed weight loss and observed malnutrition is unlikely to be attributed to the natural course of weight loss following a RYGB. However, the complete analysis of pre-ER is limited by the accessibility of pre-RYGB data. For most patients in this cohort the underlying factors precipitating chronic malnutrition years after a RYGB remains unknown. At the six-months pre-ER, patients had been medically managed for their malnutrition, with most patients receiving supplemental nutrition from 6-months pre-ER until immediately prior to their ER (baseline). Four patients in the cohort, however, had documentation for being noncompliant with their supplemental nutrition regimen. Between the 6-month pre-ER time point and the baseline time interval, the overall median weight improved as patients received appropriate, although demanding, medical management of their malnutrition. To alleviate the daily demands that medical management of malnutrition places on patients, the patients reviewed in this study underwent an ER to alter their Roux-en-Y anatomy to increase their absorptive surface area. Following the ER, most patients did not observe a significant improvement of their weight or BMI following the ER of their RYGB. Two out of the 17 patients reviewed were no longer malnourished and solely on PO nutrition at 6 months post-ER. While drastic improvement in the malnutrition status was not universally observed, continued worsening of the patient’s SPCM nutrition status and weight loss was blunted following the ER procedure. Following ER, patients established a new baseline weight as patients made attempts to reduce supplemental nutrition requirements (for example, at baseline, 8 patients were dependent on TPN, while post-ER, 3 of those 8 patients were liberated and able to be sustained on oral nutrition exclusively). Overall, the median weight was observed to stabilize between the 6-month post and 12-month post-ER time period, halting the patient’s trend in weight decline. As such, this investigation revealed that ER of RYGB may halt progressive weight loss and liberate patients from TF or TPN.

While most patients did not witness an improvement in their underlying malnutrition status, the liberation off TPN for several patients undergoing an ER cannot be understated. In this study 3 out of 8 patients (37.5%) successfully transitioned from TPN to PO nutrition. In addition to providing enormous improvements in quality of life, ER successfully mitigated the risk that accompanies using TPN in the long term. Common complications of chronic TPN use include infection, hyper and hypo glucose levels, as well as liver dysfunction (15). In the case of infection, it has been well documented that chronic use of TPN increases one risk for fungal bloodstream infections, particularly candidemia (16). Therefore, any intervention with a low risk profile that can safely transition a patient from TPN to PO nutrition is worth consideration to improve quality of life and mitigate complications seen in long term TPN use.

While most patients did not experience dramatic improvements in their laboratory markers of malnutrition, laboratory indicators of nutritional status also did not worsen throughout the period of the study (**Supplementary Table 1**, see **supplementary materials**). Throughout this study, patient’s laboratory nutritional value remained stable. This is also true in the case of patients that were initially receiving TPN and were transitioned to PO. The absence of worsening laboratory markers lends further support that ER is a safe procedure in experienced hands.

Importantly, there was not a single documented adverse event, immediate or long-term, in any of the patients followed throughout the duration of this retrospective study. Without any adverse complications, ER presents itself as a safe and viable alternative to surgical interventions, where complications following surgical RYGB reversals are frequent in the setting of SPCM (8). As such, ER may be a preferred intervention in patients with RY anatomy in the setting of SPCM that are not surgical candidates or have an aversion to further surgical interventions.

With the rising increase in bariatric surgeries performed each year, paralleling the continued annual rise in obesity, even modest comorbidity rates following bariatric surgeries are inevitably bound to impact a significant number of patients that will require subsequent interventions to improve their resulting comorbidities (17). However, even when a reversal is made surgically, the targeted symptoms do not always resolve (18). Similar to the ER performed in this study, malnutrition is the most common indication for a bariatric surgery to reverse RYGB anatomy following failure of conservative treatment (19). Bariatric and endoscopic reversals carry significantly different safety profiles. Compared to an ER a surgical RYGB is much more technically challenging, and therefore fraught with an increased risk for perioperative complications (20). Although, an ER of a RYGB still requires specialized training and is performed by an advanced endoscopists with additional training than most gastroenterologists. In the present study, ER of an RYGB reported no comorbidities following the procedure. In contrast, surgical RYGB reversal reports of persistent abdominal pain have been reported in 6.8% of patients and GERD was reported to occur in 10.2% of patients undergoing a surgical RYGB reversal (19). While there was significant morbidity following surgical RYGB reversal, neither surgical nor ER of a RYGB has had any reported mortality (19). However, in both ER and surgical RYGB reversals, many patients are lost to follow up (7). Compared to the minimal improvement in weight for most patients undergoing an ER in this study, patients undergoing a surgical RYGB reversal experienced an overall increase in weight regain in 28.8% (19). When comparing an ER of a RYGB, a surgical reversal carries with it a greater success rate in mitigating a patient’s malnutrition due to RYGB anatomy, although carries with it significant comorbidity risk. Given that patients undergoing a RYGB reversal due to malnutrition are likely to be poor surgical candidates for a surgery that already carries high risk of complication, ER may at best serve as an alternative to surgical intervention with the potential to resolve the underlying malnutrition and may otherwise serve as a bridge bariatric surgery in the management of these patient’s malnutrition. Given the minimally invasive and low risk of complication, ER maybe an ideal intervention in this patient population.

While this study examined endoscopic reversals at a single academic institution over 7 years, the sample size remains small with limited power. Although, the sample size reported here is consistent with other reports of endoscopic intervention to alter RYGB anatomy. However, as ER affords patients a potential alternative to revisional bariatric surgery, further studies are ostensibly warranted to examine longer-term nutritional and medical outcomes for severe malnutrition in the setting of post RYGB surgery.

Overall, the retrospective analysis of 17 patients undergoing an ER of RYGB anatomy revealed that ER of RYGB with LAMS is a nuanced and advance technique that permits access to the gastric remnant and is safe in experienced hands. While reversal of underlying SPCM following ER was not ubiquitously observed in this study, the patient’s weight was observed to stabilize between the 6-month post and 12-month post-ER time point, halting the patient’s trend in weight decline. As such, this investigation revealed that ER appears to halt further weight loss and macro- and micro- nutrient deficiencies. Considering that nearly 1/3 of patients that undergo a surgical RYGB reversal will experience a serious complication, alternative less invasive techniques to revert the Roux-en-Y anatomy are warranted (8). ER may be a viable alternative to surgical reversal of RYGB or may serve as a bridge to surgical reversal after failure of conservative management of SPCM.

## Data availability statement

The raw data supporting the conclusions of this article will be made available by the authors, without undue reservation.

## Ethics statement

The studies involving human participants were reviewed and approved by University of Minnesota The Institutional Review Board. Written informed consent for participation was not required for this study in accordance with the national legislation and the institutional requirements.

## Author contributions

ND and AS contributed to the design and implementation of the research, to the analysis of the results and to the writing of the manuscript; these authors contributed equally to this work. NM performed analytical methods. EW and SA helped shape the research, analysis, interpretation, and commented on the manuscript. All authors contributed to the article and approved the submitted version.
