# Supplementary material for: Endoscopic reversal of roux-en-Y gastric bypass prevents worsening of nutritional outcomes in patients with severe malnutrition
Source: Front Gastroenterol (Lausanne). 2023 Jul 14;2:1212844. doi: 10.3389/fgstr.2023.1212844 (PMC12952391; doi:10.3389/fgstr.2023.1212844)
Supplement: Supplementary file 1 [file Table_1.docx]

**Supplementary Material**

*Table 1:* Patient lab values representing micro and macro nutritional state. Results analyzed using a Friedman’s test comparing medians and interquartile ranges of collected values at baseline (immediately prior to ER reversal) and 6 months after ER reversal.

|  | N Pre | N Post | N Pre-Post | Pre Median (IQR) | Post Median (IQR) | Pre-Post Median (IQR) | p-Value |
| --- | --- | --- | --- | --- | --- | --- | --- |
| Albumin | 16 | 14 | 14 | 2.5 (2.25, 2.95) | 2.4 (1.92, 3.05) | -0.15 (-0.38, 0.7) | 0.753 |
| Prealbumin | 9 | 7 | 5 | 14 (10, 15) | 14 (8, 16) | 0 (-7, 2.6) | 0.584 |
| Vitamin D | 9 | 6 | 5 | 14 (13, 18) | 24.5 (14.5, 27.75) | 1 (-15, 6) | 0.812 |
| Calcium | 16 | 15 | 15 | 8.6 (7.9, 8.62) | 8.5 (8, 8.8) | -0.2 (-0.55, 0.4) | 0.451 |
| Magnesium | 14 | 12 | 10 | 1.9 (1.8, 2.1) | 1.8 (1.6, 1.95) | 0.1 (-0.15, 0.1) | 0.634 |
| Phosphorus | 13 | 11 | 9 | 3.4 (3.2, 4.2) | 2.7 (2.3, 3.25) | 1 (-0.5, 1.2) | 0.236 |
| PTH | 7 | 2 | 1 | 87 (44.5, 117) | 100 (93, 107) | 44 (44, 44) | - |
| Vitamin B1 | 8 | 3 | 2 | 141.5 (104, 152.5) | 176 (109, 192.5) | -74.5 (-98.25, -50.75) | 0.5 |
| Folate | 7 | 5 | 3 | 6.3 (5.4, 12.7) | 10.6 (6.5, 11.8) | -0.3 (-2.15, 3.35) | >0.99 |
| Vitamin B12 | 8 | 7 | 4 | 1405 (1038.5, 1833.5) | 1271 (731, 1422) | 491.5 (375.25, 533.25) | 0.125 |
| Vitamin A | 6 | 4 | 2 | 0.22 (0.14, 0.31) | 0.29 (0.25, 0.34) | -0.1 (-0.12, -0.08) | 0.5 |
| Vitamin E | 5 | 2 | 1 | 5.6 (5.5, 6.6) | 9.9 (8.9, 10.9) | -5.3 (-5.3, -5.3) | - |
| Zinc | 10 | 6 | 5 | 58.5 (55, 74.68) | 56.45 (48.48, 61.5) | 9 (-2, 16.4) | 0.312 |
| Transferrin | 5 | 4 | 2 | 178 (162, 338) | 196.5 (147.75, 250) | 47.5 (37.75, 57.25) | 0.5 |
| Copper | 9 | 3 | 3 | 104.3 (77, 121) | 106 (104.5, 114.5) | 9.1 (3.55, 23.55) | 0.5 |
